# Elucidation on Predominant Pathways Involved in the Differentiation and Mineralization of Odontoblast-Like Cells by Selective Blockade of Mitogen-Activated Protein Kinases

**DOI:** 10.1155/2018/2370438

**Published:** 2018-02-20

**Authors:** Jia Tang, Takashi Saito

**Affiliations:** ^1^Division of Biochemistry, Department of Oral Biology, School of Dentistry, Health Sciences University of Hokkaido, Hokkaido, Japan; ^2^Division of Clinical Cariology and Endodontology, Department of Oral Rehabilitation, School of Dentistry, Health Sciences University of Hokkaido, Hokkaido, Japan

## Abstract

**Aim:**

To analyze the effect of three mitogen-activated protein kinase (MAPK) inhibitors, namely, SB202190 (p38 inhibitor), SP600125 (JNK inhibitor), and PD98059 (ERK inhibitor) in Dex-stimulated MDPC-23 cell differentiation and mineralization.

**Methods:**

Experiment was divided into five groups, control (cells without Dex and inhibitors treatment), Dex (cells with Dex treatment but without inhibitors), Dex + SB202190, Dex + SP600125, and Dex + PD98059. Cell differentiation was assessed by alkaline phosphatase (ALP) activity assay and real time RT-PCR. Cell mineralization was investigated by alizarin red staining.

**Results:**

Exposure to SB202190 (20 *μ*M) significantly decreased the mineral deposition in Dex-treated cells as demonstrated by alizarin red staining. Treatment of SP600125 (20 *μ*M) attenuated the mineralization as well, albeit at a lower degree as compared to SB202190 (20 *μ*M). Similarly, SB202190 (20 *μ*M) completely abrogated the ALP activity stimulated by Dex at six days in culture, while no changes were observed with regard to ALP activity in SP600125 (20 *μ*M) and PD98059 (20 *μ*M) treated cells. The upregulation of bone sialoprotein (BSP), ALP, and osteopontin (OPN) in Dex challenged cells was completely inhibited by SB202190.

**Conclusion:**

Blockade of p38-MAPK signaling pathway resulted in significant inhibition of ALP activity, mineralization, and downregulation of osteogenic markers. The data implicated that p38 signaling pathway plays a critical role in the regulation of MDPC-23 cells differentiation and mineralization.

## 1. Introduction

Both bone and dentine are mineralized tissues, meaning their formation is defined by mineral crystal deposition onto organic matrix (mostly type I collagen) produced by osteoblasts or odontoblasts. Disorders that interrupt the normal mineralization process of the two tissues lead to abnormal phenotype, which seriously impair the life quality of people. For instance, osteogenesis imperfecta (OI) is a typical bone disease caused by the mutations in type I collagen; the patients tend to suffer bone fractures when subjected to only minor trauma. Another example is rickets, a defective mineralization of bone due to the lack of phosphates at the epiphyseal growth plate and mineralizing bone surfaces [1]. On the other hand, unlike bone, dentine becomes accessible to external environment with increase of time, which put it at higher risk of bacterial infection or injury. As the sensory center-dental pulp is just beneath dentine, loss of dentine not only destroys the integrity of a tooth but also deprived the pulp of a complete and healthy structural support. Therefore, elucidation on the underlying mechanism during the process of dentine mineralization is of profound importance for the development of novel dentin regeneration reagents.

Mitogen-activated protein kinases (MAPKs) are a family of conserved serine/threonine protein kinases, which contribute to a variety of cellular activities, such as proliferation [2], differentiation, migration, apoptosis, senescence [3], and stress response [4]. Typical MAPKs members include extracellular signal regulated kinase (ERK1/2 or p44/42), c-Jun N-terminal kinases 1-3 (JNK1-3), and p38 isoforms (p38*α*, *β*, *γ*, and *δ*). Since its discovery nearly three decades ago [5], MAPKs have been revealed as key players in osteoblast and odontoblast commitment and differentiation [6–9]. Based on the findings, it is thus proposed by researchers that targeting the MAPKs may offer a novel therapeutic approach for regeneration of hard tissue [10, 11].

Under the* in vitro* condition, osteoblast or odontoblast-like cells do not spontaneously undergo mineralization in the absence of any extra induction factors. When grown in the presence of synthetic glucocorticoid dexamethasone (Dex), ascorbic acid (AA), and *β*-glycerophosphate (*β*-GP), cells differentiate and produce mineralizing nodules over time [12]. Specifically, AA increases the production of collagen matrix [13]; *β*-GP acts as a source of inorganic phosphate ions [14]; Dex, a synthetic glucocorticoid, exhibits potent osteogenic and mitogenic effect [15]. In the preliminary study using the three reagents, we observed that Dex (10 nM, 100 nM) accelerated mineralization of MDPC-23 cell in the presence of *β*-GP (10 mM). The potent influence of Dex in the mineralization of MDPC-23 cell prompted us to study the potential signaling pathways that might be involved. To elucidate the effect of the three MAPKs in the differentiation and mineralization process stimulated by Dex, we used commercially available pharmacological inhibitors specific to p38 (SB202190), JNK (SP600125), and ERK (PD98059) to treat the cells prior to the addition of Dex stimulant. Cell differentiation and mineralization were analyzed.

## 2. Materials and Methods

### 2.1. Cell Culture

Rat odontoblast-like cell line (MDPC-23 cell) [16] was generously provided by Professor Jacques E. Nör (University of Michigan). The cells are cultured in Dulbecco's modified eagle's medium (DMEM, D5796, Sigma) supplemented with 5% heat-inactivated fetal bovine serum (FBS, 10270-106, Gibco) at 37°C in a humidified atmosphere and 5% CO_2_. Cells were plated at the density of 1 × 10^4^/mL and 1.25 × 10^4^/mL in 24-well (Tissue culture treated polystyrene, Iwaki) and 12-well plates (Tissue culture treated polystyrene, Falcon), respectively. Mineralization reagent including *β*-GP (10 mM) (191-02042, Wako), AA (50 *μ*g/mL) (013-19641, Wako), and Dex (100 nM) (D2915, Sigma) in DMEM (5% FBS) was added on day five, when cells reached confluence.

### 2.2. Mineralization Assay

To investigate the calcific deposition in response to three MAP kinase inhibitors, MDPC-23 cell were inoculated in 24-well plate at the density of 1 × 10^4^/mL and cultured for five days in DMEM supplemented with 5% FBS (*n* = 3). On day five, cells were challenged with the three inhibitors (SB202190, SP600125, and PD98059) (Cell Signaling Technology) for 2 h in serum free DMEM. Inhibitors containing media were replaced with mineralization inducing media including *β*-GP, AA, and Dex. Control wells represent cells treated by both *β*-GP and AA, but without Dex. On day eight, the mineralization was observed by alizarin red staining and quantified using cetylpyridinium chloride (CPC) (C0732-100G, Sigma-Aldrich) extraction method. Briefly, cell monolayer was gently washed by phosphate-buffered saline (PBS) (10010049, Gibco) and fixed in 10% formalin neutral buffer solution (060-01667, Wako) at room temperature for 20 min. Afterwards, the monolayer was rinsed by distilled water once prior to staining by 200 *μ*L alizarin red s (1%, w/v, pH 4.1 in water) (011-01192, Wako) per well. The cells were incubated with alizarin red s solution at room temperature for about 5 min. Extra dye was discarded, and the wells were washed quickly with distilled water for four to five times and after that washed with distilled water for 2 h with gentle shaking until the water became transparent. The mineralization nodules were visualized by an inverted camera (Canon) in a digital photograph system (Funakoshi). For quantification of staining, 800 *μ*L CPC (10%, w/v, in water) was poured to each well, and the plates were incubated at 37°C for 2 h. After incubation, the transparent CPC solution turned into purple and was transferred into 96-well plate (200 *μ*L/well) for absorbance reading at 570 nm.

### 2.3. ALP Activity

The cells were maintained in 12-well plate at the initial seeding concentration of 1.25 × 10^4^/mL (2 mL media/well) for five days. On day five, the three inhibitors were added to cells for 2 h. After incubation with the inhibitors, media were aspirated and replaced by mineralization inducing media (*β*-GP, AA, and Dex). Cells were cultured for another day and lysed by 0.1% Triton-X-100 (T8787, Sigma) and sonicated on ice for 10 min at day six. The cell lysates were centrifuged and supernatant was collected for ALP activity assay and protein quantification. Specifically, before conducting the ALP assay (LabAssay™ ALP, Code number 291-58601, Wako), the collected supernatant was diluted by 100 times in ultrapure water. In parallel, standard solution was prepared by series dilution of 0.5 mmol/L* p*-nitrophenol (0, 0.0625, 0.125, 0.25, and 0.5 mmol/L). The reaction mixture was constituted by 20 *μ*L of the diluted samples or standard solution and 100 *μ*L of the* p*-nitrophenylphosphate substrate. Prior to incubation, the mixture was thoroughly mixed using plate mixer for 1 min. After incubation under 37°C for 15 min, the reaction was terminated by addition of 0.2 mol/L sodium hydroxide. Absorbance was read at 405 nm. The calculated ALP activity was divided by protein concentration, which was quantified using Pierce BCA assay kit, to avoid the influence of protein amount variation.

### 2.4. Real Time RT-PCR

Cells were seeded into 12-well plate at the initial density of 1.25 × 10^4^/mL. On day five, cells were exposed to the three inhibitors (20 *μ*M) for 2 h; mineralization reagent was added after inhibition treatment on the same day. On day seven, total RNA was purified from cells in 12-well plate (*n* = 3) using Trizol® reagent (Invitrogen, Carlsbad, CA, USA) according to manufacturer's instruction. The concentration of RNA was determined using a Nanodrop 2000 spectrophotometer (Thermo Fisher Scientific, Wilmington, DE, USA). One microgram purified RNA was treated with RNase inhibitor and reverse transcribed into complementary DNA (cDNA) in a 20 *μ*L reaction system by the Moloney Murine Leukemia Virus (M-MLV) reverse transcriptase (1 *μ*g RNA; DNase and RNase free water; 1 *μ*L Oligo (dT) (18418-012, Invitrogen); 4 *μ*L dNTP mixture (2.5 mM each, 4030, TaKaRa); 1 *μ*L RNase inhibitors (40 U/*μ*L) (Cat#2313A, TaKaRa); 4 *μ*L 5x first strand buffer (Invitrogen, Carlsbad, CA, USA); 2 *μ*L 0.1 M dithiothreitol (DTT, Invitrogen); 1 *μ*L Moloney Murine Leukemia Virus (M-MLV) enzyme (200 U/*μ*L) (REF 28025-013, Invitrogen)). Following the synthesis of cDNA, real time PCR was carried out in a LightCycler Nano® (Roche) system using FastStart Essential DNA Probes Master (2x) (Roche). Target gene expression was normalized by housekeeping gene *β*-actin. The mean value for the control group was taken to be 100% of mRNA expression and served as a reference. The detailed information for primers and real time PCR reaction conditions are listed in Table 1. The SYBR green amplification reaction consisted in an initial denaturation of 10 min at 95°C, followed by 50 cycles of 15 s at 95°C (denaturation), 30 s at annealing temperature (see Table 1 for each set of primer), and 30 s at 72°C (extension). The 2^−ΔΔCt^ method was used to calculate relative gene expression.

### 2.5. Statistical Analysis

Data are presented as average ± standard deviation (SD). The statistical analysis of differences among the groups was analyzed by post hoc Tukey HSD test at a 5% level of significance.

## 3. Results

### 3.1. Mineralization Assay

**Figure 1:**
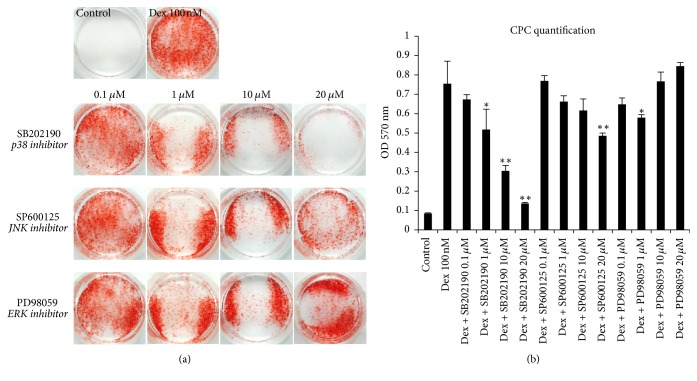
*Effects of blockade of p38, JNK, or ERK activation on mineralizing nodules formation.* Cells were cultured in control medium (*β*-GP and AA) or mineralization medium (*β*-GP, AA, and Dex) or mineralization medium supplemented with SB202190, SP600125, or PD98059. Alizarin red staining was performed on day eight as described under Materials and Methods Section 2.2. Addition of Dex significantly accelerated mineralization, as staining was hardly observable in control containing only *β*-GP and AA (a). SB202190 inhibits mineralization in a concentration-dependent manner ((a) and (b)). SP600125 slightly attenuates the mineralization at the concentration of 20 *μ*M. PD98059 (1 *μ*M) marginally decreased the mineralization, while PD98059 in the other concentration groups did not impact it (^*∗*^*p* < 0.05; ^*∗∗*^*p* < 0.01; differences were given out as compared to Dex group).

The potential effect of the three inhibitors on calcific deposition in MDPC-23 cell was explored using alizarin red staining. The staining photograph (Figure 1(a)) showed that 2 h exposure to SB202190, the p38 inhibitor, leads to a significant reduction of mineral deposition stimulated by Dex; the mineralization of cells treated by SB202190 decreased in a concentration-dependent manner (Figure 1(b)). Particularly, a concentration of 20 *μ*M almost completely blocked the mineral deposition of cells (optical density 0.14 ± 0.01 versus 0.09 ± 0.00 of control, *p* < 0.01). On the other hand, although SP600125 exhibited mineralization inhibition activity, the influence was much weaker as compared to SB202190: SP600125 (20 *μ*M) (optical density: 0.49 ± 0.02) only inhibits 35% of the mineralization induced by Dex (0.76 ± 0.12). With regard to the cells treated by PD98059, slight decrease of mineralization was observed in the group of 1 *μ*M (0.58 ± 0.02 versus 0.76 ± 0.12 in Dex group, *p* < 0.01). No statistical differences were found between Dex group and the other PD98059 (0.1, 10, and 20 *μ*M) groups.

### 3.2. ALP Activity

**Figure 2:**
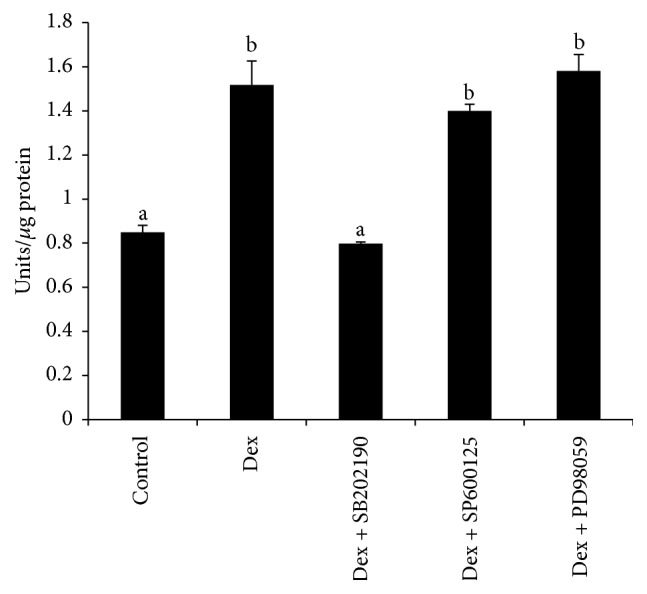
*Inhibition of ALP activity by SB202190 in MDPC-23 cell*. ALP activity on day six was determined as described under Materials and Methods. The results represent average ± SD of cultures (*n* = 4). ALP activity in cultured treated with SP600125 (20 *μ*M) or PD98059 (20 *μ*M) alone was similar to those of Dex group. On the contrary, treatment of cells using SB202190 completely inhibited the ALP activity stimulated by Dex (a-b indicate significant differences between different characters, *p* < 0.01).

The ALP activity was markedly augmented by Dex at the concentration of 100 nM (1.52 ± 0.11 units/*μ*g protein versus 0.85 ± 0.03 units/*μ*g protein of control, *p* < 0.01) (Figure 2). This upregulation of was completely hindered by the addition of SB202190 (20 *μ*M) (0.80 ± 0.01 units/*μ*g protein). The addition of SP600125 (1.40  ±  0.03 units/*μ*g protein) or PD98059 (1.58  ±  0.07 units/*μ*g protein) did not impact the ALP activity stimulated by Dex.

### 3.3. Real Time RT-PCR

**Figure 3:**
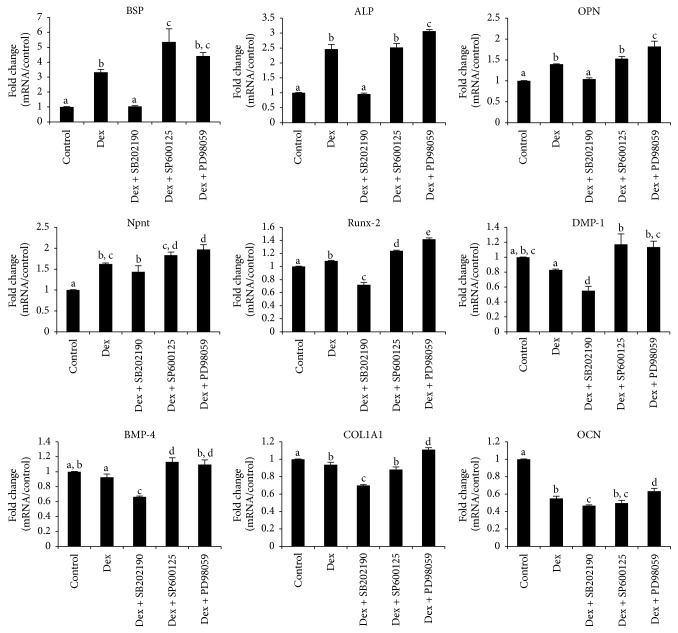
*Gene expression profile of osteogenesis related markers in culture containing SB202190, SP600125, or PD98059*. MDPC-23 cells were cultured as described in Materials and Methods. Total RNA was isolated on day seven and analyzed by reverse-transcription PCR with the indicated primers illustrated in Table 1. Control means cells cultured in the presence of *β*-GP and AA, without Dex or any inhibitors. Dex group means cells maintained in *β*-GP, AA, and Dex, without any inhibitors. The concentration for the three inhibitors was unified to be 20 *μ*M (a–e indicate significant differences between different characters in each panel, *p* < 0.01).

Gene expression of BSP, ALP, OPN, nephronectin (Npnt), runt-related transcription factor 2 (Runx-2), dentine matrix protein-1 (DMP-1), bone morphogenetic protein-4 (BMP-4), collagen I (COL-1), and osteocalcin (OCN) were assessed by real time RT-PCR (Figure 3). Among those, BSP (3.33 ± 0.19-fold), ALP (2.47 ± 0.15-fold), and OPN (1.40 ± 0.01-fold) were significantly promoted by Dex; concomitantly, the upregulation of the three genes was completely impeded by SB202190 (20 *μ*M). On the contrary, exposure to SP600125 (20 *μ*M) further boosted the expression of BSP (5.36 ± 0.87-fold), while it did not affect ALP and OPN expression as compared to Dex group. Moreover, PD98059 (20 *μ*M) slightly upregulated ALP (3.06 ± 0.05-fold) and OPN (1.82 ± 0.13-fold) compared to Dex. There was no change in relation to the expression of BSP after inclusion of PD98059. Expression of Npnt (a newly discovered extracellular matrix protein) was significantly promoted by Dex (1.62 ± 0.03-fold). SB202190 and SP600125 did not affect the expression of Npnt, but PD98059 slightly enhanced its expression (1.97 ± 0.11-fold). mRNA expression of Runx-2 was marginally higher in Dex group (1.09  ±  0.01-fold) than control. Similarly, SB202190 repressed its expression while both SP600125 and PD98059 upregulated it compared to Dex group. For the rest of four genes, all were downregulated by Dex treatment, remarkably, and OCN expression level was inhibited to nearly 50% in Dex group. The expression profile of the four genes modulated by the three inhibitors was similar, with SB202190 downregulating and the other two upregulating the expression.

**Figure 4:**
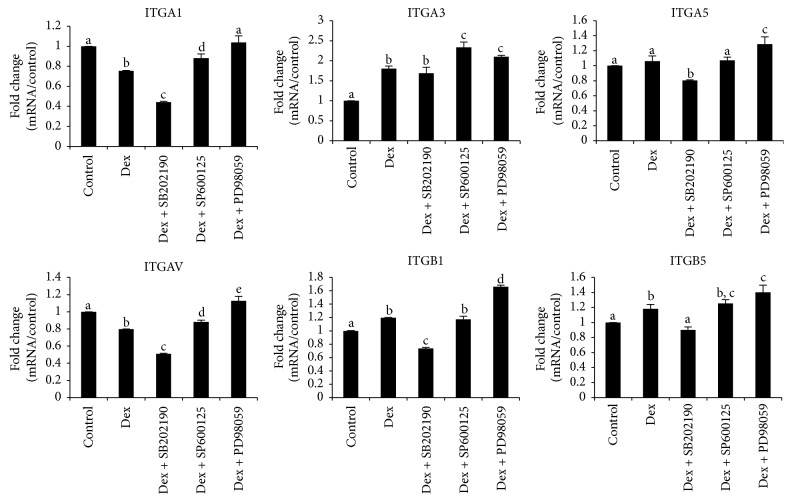
*Gene expression profile of integrins in culture containing SB202190, SP600125, or PD98059.* MDPC-23 cells were cultured in the same way as Figure 3. Total RNA was isolated on day seven and analyzed by reverse-transcription PCR with the indicated primers illustrated in Table 1. Control means cells cultured in the presence of *β*-GP and AA, without Dex or any inhibitors. Dex group means cells maintained in *β*-GP, AA, and Dex, without any inhibitors. The concentration for the three inhibitors was unified to be 20 *μ*M (a–e indicate significant differences between different characters in each panel, *p* < 0.01 except for *p* < 0.05 between Dex and Dex + PD98059 in ITGA3 panel; *p* < 0.05 between Dex and Dex + SP600125 in ITGAV panel; *p* < 0.05 between control and Dex in ITGB5 panel).

To further characterize the mRNA expression of integrins, the well-established cell surface receptors for a number of extracellular proteins, total six types of integrins (integrin alpha 1 (ITGA1), integrin alpha 3 (ITGA3), integrin alpha 5 (ITGA5), integrin alpha v (ITGAV); integrin beta 1 (ITGB1) and integrin beta 5 (ITGB5)) were evaluated by real time RT-PCR (Figure 4). Among the four alpha integrins, ITGA3 was markedly enhanced by Dex (1.80 ± 0.06 fold); the upregulation was not altered by incorporation of SB202190 (1.70 ± 0.15-fold) but was further strengthened by SP600125 (2.33 ± 0.14-fold) and PD98059 (2.10 ± 0.04-fold). Expression of both ITGA1 (0.75 ± 0.00-fold) and ITGAV (0.80 ± 0.00-fold) was marginally retarded by Dex, while that of ITGA5 was unchanged in Dex group compared to control. Two beta integrins (ITGB1 and ITGB5) were slightly promoted by Dex. In agreement with the above noted trend, SB202190 inhibited the expression of ITGA1, ITGA5, ITGAV, ITGB1, and ITGB5 while SP600125 and PD98059 enhanced them.

## 4. Discussion

Although the activation of MAPKs has been associated with osteo/odontoblast differentiation and mineralization, it is unclear as to which pathway plays a predominant role. In the present study, to clarify the underlying signal pathways involved in the differentiation and mineralization stimulated by Dex, we used three MAPK inhibitors to investigate their respective effects in a series of cell behavior. SB202190, a pyridinyl imidazole, is cell permeable and highly selective inhibitor of p38*α* and *β* isoforms. It shares structure similarity with another p38 inhibitor-SB203580 and is usually used as an alternative to SB203580. The specificity of SB202190 toward p38 pathway was revealed by its low half maximal inhibitory concentration (IC_50_) and failure to affect other protein kinases [17]. SP600125 is a novel selective JNK1/2/3 inhibitor, which causes inhibition of phosphorylation of c-Jun. Its IC_50_ toward JNK1/2/3 was found to be as low as 1/272 of the value on ERK2 or p38*β*, indicating SP600125 was highly specific for JNK [18]. PD98059 binds to inactive forms of MEK1 and prevents activation by upstream activators such as c-Raf [19]. In the comparative study that compares 28 types of commercially available kinases inhibitors, PD98059 stands out by its superior specificity: it did not show any inhibition activity at a concentration of 50 *μ*M, by which concentration ERK was inhibited [17]. The concentration of each inhibitor used in the present experiment was based on recommendations from the manufacturer. It was suggested that the working concentration for SB202190, SP600125, and PD98059 is 5–20 *μ*M, 25–50 *μ*M, and 5–50 *μ*M, respectively. To avoid causing cytotoxicity, we selected the lowest maximal working concentration from SB202190 (20 *μ*M) for the experiment.

Maintenance of a health pulp is a longstanding issue and of critical importance, as teeth devitalized by root canal treatment become more vulnerable and prone to structural failure over time. Odontoblasts share some common features with osteoblasts: both of them secrete extracellular matrix that undergoes mineralization [20, 21]. Nevertheless, the uniqueness of the former lies in the fact that they are trapped at the periphery of dental pulp and produce reparative dentine in case of attrition, deep caries, or injury. The implications of tooth devitalization have driven significant interest in research with regard to the development of bioactive materials that facilitate the regeneration of damaged pulp tissue by harnessing the capacity of dental pulp for self-repair. Recently, there are a large number of* in vitro* and* in vivo* studies that investigated the relevant role of p38, JNK, and ERK throughout the osteoblast/odontoblast differentiation process, from mesenchymal stem cells to fully committed anabolic osteoblast/odontoblast-like cell lines. For example, p38 phosphorylation was increased in an* in vitro* caries model established by addition of* Streptococcus mutans *in MDPC-23 cell [22]. Importantly, although ERK was activated in parallel with p38 MAPK in the differentiation of primary calvarial osteoblast, inhibition of ERK did not affect osteoblast differentiation in terms of ALP activity and mineral deposition, while inhibition of p38 significantly suppressed the ALP activity and mineralization [23]. On the other hand, JNK signal pathway was noted to be required for late stage differentiation in both MC3T3-E1 cells and primary calvarial osteoblasts, as demonstrated by a significant inhibition of OCN and BSP expression [24]. Phosphorylation of p38, JNK, and ERK was increased by fucoidan, a type of polysaccharide, during the differentiation process of human alveolar bone marrow mesenchymal stem cells, but it was found that osteogenic differentiation induced by fucoidan was inhibited by SP600125 and PD98059 but not SB203580 (another type of p38 inhibitor) [25]. More recently, a study using calcium hydroxide, the commonly used pulp capping material, in dental pulp stem cells (DPSCs) has revealed p38, JNK, and ERK are all responsible for enhanced differentiation in DPSCs [26].

Here, we showed that inhibition of p38 signaling pathway by SB202190 interfered with the differentiation and mineralization process of the MDPC-23 cell. ALP activity, an important parameter for evaluating initiation but not progression of osteoblast/odontoblast differentiation, was completely blocked by this p38 inhibitor; in comparison, the treatment of SP600125 and PD98059 did not alter the ALP activity as compared to Dex group, reflecting that ALP activity upregulation stimulated by Dex was predominantly regulated via p38 signaling pathway, neither JNK nor ERK pathways.

Next, real time RT-PCR was conducted to assess multiple hard tissue forming genes. In contradiction to the results presented by Kim et al. [25], it was found that expression of BSP, ALP, and OPN, three classical osteogenesis markers, was completely inhibited in cell treated with SB202190 but not SP600125 or PD98059. The discrepancy may be caused by different cell type and stimulant used for osteogenic induction. Npnt, recently reported to be able to induce the differentiation of MDPC-23 cell into an odontoblast-like phenotype [27], was upregulated by Dex at the concentration of 100 nM to 1.6-fold more than control; its expression was unaffected by the treatment with neither SB202190 nor SP600125, but that PD98059 slightly enhanced it. The observation denoted that some other signal pathways are involved in the Dex-mediated upregulation of Npnt. Indeed, a recent work demonstrated that Wnt/*β*-catenin signaling pathway is responsible for the activation of Npnt by Wnt3a in MC3T3-E1 cell [28]. Whether the same signaling pathway is required in Dex-induced Npnt expression awaits further investigation. Marginal upregulation of Runx-2 (or core-binding factor subunit alpha-1, CBF-*α*1) was detected and that inhibition of p38 downregulated the expression, while inhibition of JNK and ERK upregulates it. This correlates well with a study by Lee et al. [29], who showed that strong induction of Runx-2 using a p38 activator (anisomycin) was blocked by the addition of SB203580 (a specific p38 inhibitor). This further demonstrated that p38 signaling pathway is positively involved in the regulation of Runx-2 expression. DMP-1, an acidic protein found in mineral phase of vertebrates and invertebrates, is a key regulatory protein of odontogenesis [30]. We found that p38 inhibition led to a significant reduction of DMP-1 expression, while JNK and ERK inhibition enhanced it. Previously, it was reported that downregulation of DMP-1 in KN-3 cell (rat incisors dental papilla derived cell line) initiated by interferon-*γ* (IFN-*γ*), a proinflammatory cytokine, was achieved through the phosphorylation of p38 MAPK [31]. BMP-4 is a potent inducer for odontoblast differentiation [32], it is shown that there was no change in terms of the gene expression for DMP-1 and BMP-4 upon treatment by Dex. Since BSP, ALP, and OPN are well-established osteogenic factors, the data denoted Dex may induce the transdifferentiation of MDPC-23 cells into osteoblast. Similarly, the inhibition of BMP-4 and COL1A1 by SB202190 indicated that gene expression of both may be partially regulated via the p38 signaling pathway. Indeed, Tan et al. [33] found that the upregulation of BMP-4 activated by CCN3 was abrogated by p38 and JNK inhibitors. COL1A1, the gene encoding alpha chain of type I collagen, was inhibited by exposure to SB202190 and promoted by PD98059, denoting that this gene is regulated by p38 and ERK signaling pathway and unaffected by JNK. Finally, the OCN expression was significantly downregulated by Dex, although there was slight reduction of expression in SB202190 group, and the difference was not evident, suggesting that some other signaling pathways might be involved.

As cell matrix is regulated to a large extent by surface receptors such as integrin, we further expanded our investigation to analyze the gene expression of integrin stimulated by Dex and MAPK inhibitors. Thus far, there are 24 known integrins. We characterized six subtypes of integrin (ITGA1, ITGA3, ITGA5, and ITGAV and ITGB1 and ITGB5) by real time RT-PCR. ITGB1, a ubiquitously expressed integrin, was promoted. Indeed,* in vivo* ITGB1 knockout mice were found to exhibit delayed eruption of molars, indicating that it was indispensable for the tooth development [34]. Among the four alpha integrins, only ITGA3 was significantly promoted by Dex, and SB202190 did not impact the expression of ITGA3, denoting that p38 is not involved in the regulation of ITGA3 expression. There was no change in terms of ITGA5 expression after exposure to Dex, but ITGA1 and ITGAV were downregulated. Furthermore, SB202190 markedly downregulates the expression of ITGA1, ITGA5, ITGAV, ITGB1, and ITGB5. In contrast, SP600125 and PD98059 significantly enhanced the expression of all the integrins. It is thus suggested that Dex induced an altered integrin expression pattern, which were further changed by the exposure to MAPK inhibitors.

Dentine undergoes continuous matrix deposition. Odontoblasts are cells that line the periphery of the pulp and responsible for dentine matrix secretion and mineralization. In the case of injury, certain signaling pathways in odontoblasts are activated by inflammatory factors to initiate wound healing process. Among various pathways inside a cell, MAPKs are a family of enzymes that are implicated in a series of processes. Here, we established the mineralization and differentiation model in MDPC-23 cell by stimulating it with Dex and identified the signaling pathways of switching odontoblasts from quiescent state to active secretion state. We selected three specific MAPKs inhibitors and clarified their effects when added to cell culture media. The results, which correlate well with previous literatures, underline a critical role of p38 in the regulation of differentiation and mineralization in MDPC-23 cell. Qin et al. examined differentiation of human dental pulp cells into odontoblasts using bone morphogenetic protein-2 (BMP-2) and detected enhanced phosphorylation of p38*α*; moreover, knock-down of this pathway inhibited ALP activity and mineralization and suppression of p38*α* attenuated odontoblastic differentiation [35]. Another comparative study of early and late stage odontoblasts suggested that p38 was intensively expressed by early stage odontoblasts and disappeared in late stage odontoblasts, denoting that it was involved in the primary dentine formation [36]. Interestingly, Yu et al. purified human dentin matrix proteins cocktail and added them to bone marrow-derived mesenchymal stem cells to find that both ERK and p38 were phosphorylated, and inhibition of this two pathways simultaneously suppressed mineralization and gene expression of osterix and DSPP [37]. It is hence should be pointed out that, due to the differences in stimulants, cell types, and experiment design, the potential effects of p38, JNK, and ERK reported in literatures are not always the same, sometimes even contradictory. One should thus be careful to interpret the data in published literatures.

In the present work, we used Dex as a mineralization inducer to establish an* in vitro* differentiation model in MDPC-23 cell and compared the effects of three specific inhibitors to p38, JNK, and ERK. Despite the limitations of the current experiment, it is suggested that p38 signaling pathway emerges to be an important member in the regulation of differentiation and mineralization of MDPC-23 cell.

## 5. Conclusion

To summarize, analysis of the data presented by the current study demonstrated that addition of Dex into the mineralization media accelerated differentiation and mineralization in MDPC-23 cell and that inhibition of the MAPK pathways (p38, JNK, and ERK) identified SB202190, a specific p38 MAPK inhibitor completely blocked the ALP activity, expression of osteogenesis markers including BSP, ALP, and OPN, and mineralization. As shown above, there are several studies on Dex-induced signaling via MAPKs regulating osteogenic differentiation and mineralization in other cell types; however, none has reported those effects in odontoblast-like cell. Benefit from the current work provides a possible rationale for future work to use small-molecule activators toward p38 as a treating modality for induction of hard tissue formation in dental pulp lesion.

## Figures and Tables

**Table 1:** Primers information.

Gene name	Forward	Backward	Fragment size (bp)	Annealing temperature (°C)
BSP (NM_012587.2)	CTGCTTTAATCTTGCTCTG	CCATCTCCATTTTCTTCC	211	55
ALP (NM_013059.1)	GGAAGGAGGCAGGATTGACCAC	GGGCCTGGTAGTTGTTGTGAGC	338	55
OPN (NM_012881.2)	TTTCCCTGTTTCTGATGAACAGTAT	CTCTGCTTATACTCCTTGGACTGCT	228	55
Npnt (XM_008761543.1)	CTCAAAGCTGTGTGCCAACC	TTGTGGCTTGATGATCCGGG	178	59.9
Runx-2 (NM_001278484.2)	CCACAGAGCTATTAAAGTGACAGTG	AACAAACTAGGTTTAGAGTCATCAAGC	87	55
DMP-1 (NM_203493.3)	CGTTCCTCTGGGGGCTGTCC	CCGGGATCATCGCTCTGCATC	577	62
BMP-4 (NM_012827.2)	CAGGGCCAACATGTCAGGAT	TGGCGACGGCAGTTCTTATT	188	59.9
COL1A1 (NM_053304.1)	AGAATATGTATCACCAGACG	CAGCTGATTTCTCATCATAG	224	43
OCN (NM_013414.1)	AGCTCAACCCCAATTGTGAC	AGCTGTGCCGTCCATACTTT	190	55
ITGA1 (NM_030994.2)	TCAACGTTAGCCTCACCGTC	CAGGGATCGTCTCATTGGCA	396	59.9
ITGA3 (XM_003750907.2)	GAAAGGCTGACCGACGACTA	TGCGTGGTACTTGGGCATAA	108	66
ITGA5 (NM_001108118.1)	GAAGGGACGGAGTCAGTGTG	TGAATGGTGCTGCACTGGAT	127	66
ITGAV (XM_008762000.1)	ATAAAGCGCGGATGGCAAAG	CTCACCCGAAGATAGGCGAC	213	64.9
ITGB1 (NM_017022.2)	ACAAGAGTGCCGTGACAACT	AGCTTGATTCCAAGGGTCCG	325	59.9
ITGB5 (NM_147139.2)	CACGGTCCATCATCTCTCGG	CATGGAGAGGGAGAGGTCCA	281	62.8
*β*-actin (NM_031144.3)	AACCCTAAGGCCAACAGTGAAAAG	TCATGAGGTAGTCTGTGAGGT	241	53
